# The influence of heterogeneous learning ability on the evolution of cooperation

**DOI:** 10.1038/s41598-019-50451-2

**Published:** 2019-09-26

**Authors:** Xiaogang Li, Yini Geng, Chen Shen, Lei Shi

**Affiliations:** 1 School of Statistics and Mathematics, Yunnan University of Finance and Economics, Kunming, 650221 China; 2 School of Statistics and Mathematics, Shanghai Lixin University of Accounting and Finance, Shanghai, 201209 China

**Keywords:** Physics, Statistical physics, thermodynamics and nonlinear dynamics

## Abstract

In this paper, we design a simple coevolution model to investigate the role of heterogeneous learning ability on the evolution of cooperation. The model weakens the winner’s learning ability in order to keep its current advantage. Conversely, it strengthens the loser’s learning ability for increasing the chance to update its strategy. In particular, we consider this coevolutionary model separately applying to both cooperators and defectors (rule I), only cooperators (rule II), as well as only defectors (rule III) in spatial prisoner’s dilemma game. Through numerical simulations, we find that cooperation can be promoted in rule II, whereas, cooperation is hampered in rule I and rule III. We reveal its potential reason from the viewpoint of enduring and expanding periods in game dynamics. Our results thus provide a deeper understanding regarding the heterogeneous learning ability on game theory.

## Introduction

As the core of all social dilemmas, the clash between personal prosperity and collective interests (social welfare) has become the focus of research in the fields of biology, economics and sociology^1–3^. Over the past two decades, the application of evolutionary game theory is consistently proven to be the most effective and mature tool for exploring the emergence and sustainability of cooperation^4–6^, and none has received as much attention as the prisoner’s dilemma game (*PDG*)^7,8^. By ranking the four payoffs (see the methods section for details), mutual cooperation can yield the highest collective payoff, yet the best choice for selfish individuals is defection regardless of the strategy adopted by the opponent.

Based on the research of Nowak *et al*.^9^, cooperation in a selfish environment has been maintained stably and further promoted under the application of many mechanisms^10,11^. Based on the more realistic situation, many mechanisms have been also proposed^12–16^, such as asymmetric interaction^17–19^, reward (or punishment)^20,21^, social diversity^22–25^, partner selection^26^, different update rules^27,28^, mobility^29–32^, multi-strategy (loner, tit-for-tat)^33–36^, to name but a few. Besides, heterogeneity provides diversity phenomenons for evolutionary games^37–43^. For example, the heterogeneity of age that is introduced into individual payoff by constructing an age-dependent function can promote the level of cooperation among population, especially when some individuals with certain characteristics or abilities became more influential^44^. Moreover, Amaral *et al*. explored the heterogeneity of the game and found that cooperation was reborn and greatly promoted by playing mixed games (including four different classes of game) on two different networks^45^. However, contrary to the above conclusions, Perc explored the impact of heterogeneity in public goods game by setting two types of scaling factors with uniform distribution and exponential distribution, and found that strong heterogeneity did not show advantage in facilitating cooperation^46^. In addition, the experimental test found that there is almost no difference in the level of cooperation observed between the lattice and the scale-free network in the paired interactive prisoner’s dilemma^47^. More recently, the investigation about the effect of heterogeneity has the answer already: it depends on the specific definition of heterogeneity and updating rule^48^.

In this paper, we further investigate the effect of heterogeneous learning ability on the evolution of cooperation. We therefore design a simple coevolutionary model, where the winner-weaken-loser-strengthen rule was incorporated, to test its performance on cooperation. This model decreases a player’s learning ability once its payoff is no less than the average payoff of its neighbors. Conversely, the learning ability of a player is increased when its payoff is less than the average payoff of its neighbors. Particularly, we also consider this coevolutionary model separately applying to both cooperators and defectors (rule I), only cooperators (rule II), as well as only defectors (rule III) in spatial prisoner’s dilemma game. Obviously, the heterogeneity of players’ learning ability is thus introduced in these three rules by this simple coevolutionary model. Interestingly, we find that the evolution of cooperation has different performance in a heterogeneous population, where cooperation can be promoted in rule II, however, both rule I and rule III hamper the evolution of cooperation. Through our coevolutionary model, heterogeneous learning ability can be formed spontaneously no matter what rule is applied. However, heterogeneity alone can not explain the above results. We seek to its potential reason from the viewpoint of enduring and expanding periods as suggested in Wang *et al*. and Shigaki *et al*.^49,50^. Evolutionary snapshots reveal that, in rule II, cooperators have lower learning ability compared with defectors during the enduring periods, which enables the best environment for cooperation to evolve and increases the strength of network reciprocity. Whereas, in the other two rules, the situation is different, which decreases the survival chance of cooperators and lead to the easier invasion of defectors.

## Results

**Figure 1:**
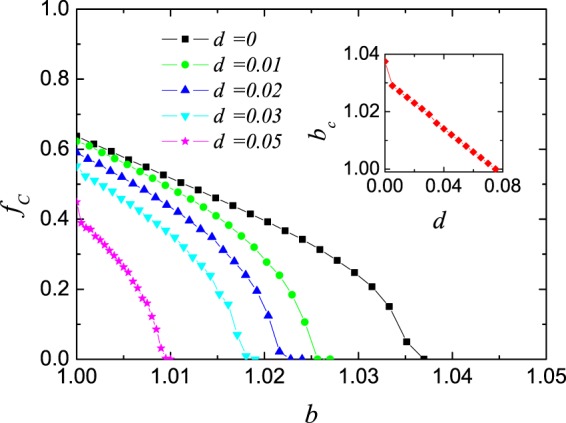
Density of cooperation *f*_*C*_ in dependence on temptation to defect *b* for *d* = 0, 0.01, 0.02, 0.03 and 0.05. Compared with the basic version of the game (*d* = 0), *f*_*C*_ declines monotonously with increasing values of *d*. The inset shows the linear negative correlation between *d* and the threshold of *b* when cooperators disappear.

We first consider the level of cooperation in dependence on the temptation to defect *b* for different values of increment *d* in Fig. 1. Compared with the basic version of the game (*d* = 0, each player can only carry out the evolution of strategy and learning ability *w* will maintain the homogeneous initialization setting throughout the game), cooperation is restrained effectively. It is obvious that cooperators can survive in smaller range of *b* with increasing values of *d*. The same phenomenon can be also observed when considering the correlation between *d* and the threshold *b*_*C*_ of cooperation vanishing in the inset. There is analogously linear negative correlation between *d* and *b*_*C*_, and *b*_*C*_ drops to 1 when *d* approaches 0.08, which means that it is harder for cooperators to survive for higher values of *d*.

**Figure 2:**
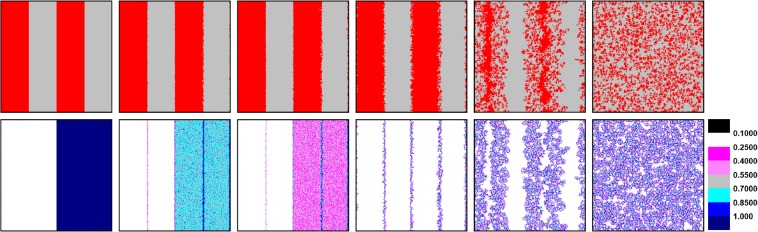
Characteristic snapshots of strategy (the top row) and learning ability (the bottom row) under specific initial distribution at 0, 10, 20, 100, 1000 and 50000 Monte Carlo steps. Initially, players are separated into four parts, where learning ability of cooperators (red) and defectors (gray) on the left and right is fixed as 0.1 and 1, respectively. The results are obtained for *d* = 0.03, *b* = 1.01 and *L* = 300.

Apparently, heterogeneous learning ability can not facilitate cooperation in rule I, which further confirms the conclusion about the diversity impact of heterogeneity on cooperation that we mentioned in above. In order to find out the key point that affects cooperative behavior, we present evolutionary snapshots of strategy (the top row) and learning ability *w* (the bottom row) at the same MC step in Fig. 2. Specifically, in the beginning, cooperators and defectors are separated into four bar-type parts, where learning ability of players on the left and right are fixed as the minimum value 0.1 and the maximum value 1, respectively. Such a setting provides us a convenient way to investigate the coevolution of strategy and learning ability evolving for players with extreme *w*.

Initially, cooperators and defectors on the boundary adopt strategies from each other. And the rougher interface signifies the strategy learning behavior is more acute between players with higher *w* (players on the right). During this process, learning ability of players on the boundary changes slowly for their frequent learning behavior. Particularly, players on the left (right) boundary tend to increase (decrease) the value of *w* in account of their initial learning ability setting. In contrast, players regardless of cooperators or defectors within the cluster prefer to decrease their learning ability until *w* reaches its minimum value 0.1 for they always have the same payoffs with environment, in which inside players on the left keep their initial lowest learning ability 0.1 unchanged. The system is relatively stable up to 100 MC steps, where the value of *w* of players inside the cluster equals to 0.1, while players on the boundary appear heterogeneity on learning ability. In this case, most border area is occupied by cooperators for their lower payoffs, which makes it convenient to separate cooperators by invading of defectors subsequently and indicates the start of enduring (END) period mentioned in^49^. The rapid downfall of cooperation stops at the domination of defectors, where cooperators in the cluster can gain the highest payoffs and defectors can hardly obtain payoffs for being surrounded by players with the same decision. Naturally, the conditions of cooperators and defectors shift and switch to the expanding (EXP) period, so that cooperators can survive ultimately. However, it is also hard for cooperators to spread widely for their higher learning ability.

As we can find in Fig. 2, cooperators on the boundary are easy to enhance their learning ability under the exploitation of defectors. Even though they reverse the situation slightly later, there are still some cooperators with higher learning ability existing in the population. Thus, cooperators can survive by enduring defectors’ invasion successfully, but it is hard for them to prevail. In order to have an embedded understanding of learning ability, we further employ two extension rules, where only cooperators (defectors) are allowed to evolve their learning ability *w* through iteration process.

**Figure 3:**
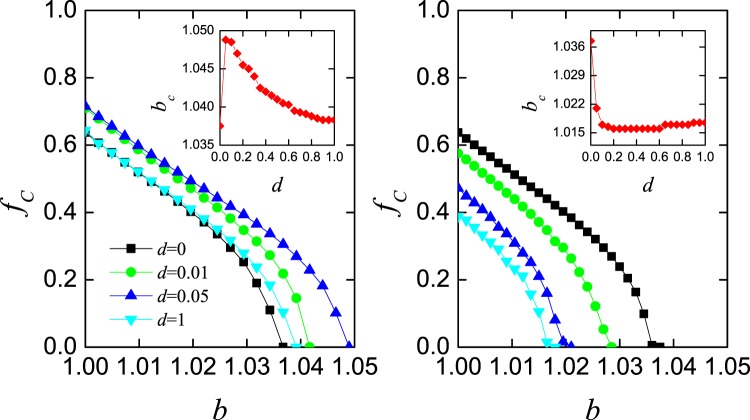
Density of cooperation *f*_*C*_ in dependence on *b* for *d* = 0, 0.01, 0.05 and 1 for two extension rules, where the coevolutionary behavior can only happen on cooperators (the left panel, rule II) or defectors (the right panel, rule III). The inset shows the correlation between *d* and the threshold of *b* when cooperators disappear.

For the above extension rules, we show how density of cooperation *f*_*C*_ varies in dependence on the temptation to defect *b* for four different values of increment *d*. In Fig. 3, the left and right panels depict the cases of only cooperators and defectors evolving their learning ability, respectively. Intuitively, cooperation can be facilitated if only cooperators have coevolutionary behavior, whereas the phenomenon in the right panel is generally consistent with the results in Fig. 1, where cooperation is suppressed for increasing *d*. In addition, the positive effect in the left panel does not enhance continuously with increasing values of *d* and meets its optimal level at *d* = 0.05. As shown in the inset of depicting the correlation between *d* and *b*_*C*_, a peak arises at *d* = 0.05, whereafter *b*_*C*_ declines with increasing *d* and always remains larger than the threshold in traditional version. As for the situation of only defectors evolving learning ability, the threshold of cooperation vanishing is pretty small and almost the same for most values of *d* (as shown in the right inset).

**Figure 4:**
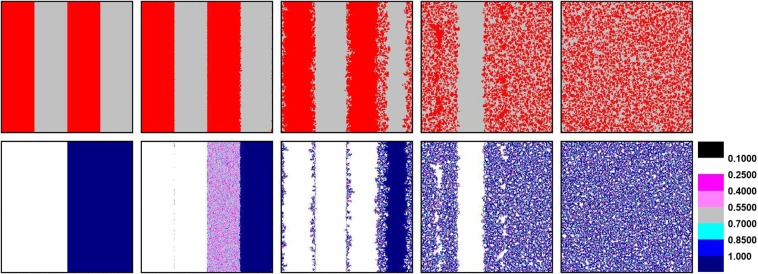
Characteristic snapshot of strategy (the top row) and learning ability (the bottom row) for rule II, where only cooperators have the right to evolve their learning ability under specific initial distribution at 0, 10, 200, 900 and 50000 MC steps. The implication of each colors is consistent with the meaning in Fig. 2. The results are obtained for *d* = 0.05, *b* = 1.02 and *L* = 300.

**Figure 5:**
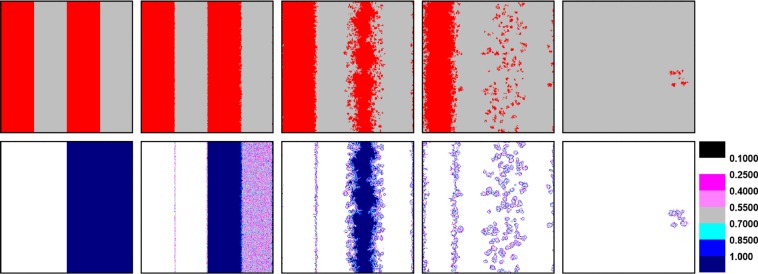
Characteristic snapshot of strategy (the top row) and learning ability (the bottom row) for rule III, where only defectors have the right to evolve their learning ability under specific initial distribution. The parameters are consistent with those in Fig. 4.

Likewise the initial distribution setting in Fig. 2, we employ characteristic snapshots to analyze the evolution of strategy and learning ability for the above two extension rules in Figs 4 and 5. We first consider the evolution in Fig. 4, where only cooperators have the right to evolve their learning ability. The evolution processes are similar for players with *w* = 0.1 between Figs 2 and 4. In detail, the value of *w* of players in the cluster remains the same, while a few cooperators on the boundary can enhance their learning ability. However, for players on the right, defectors with changeless *w* = 1 prefer to adopt strategies from cooperators with lower *w* through evolution, so that cooperators can collapse the field of defectors effectively. Conversely in Fig. 5, cooperators with *w* = 1 are exploited by defectors, whose learning ability has already reached the minimum value *w* = 0.1 through evolution. In this case, defectors with lower learning ability and higher payoff disintegrate cooperation camp rapidly.

Comparing above evolution processes of two extension rules, cooperators with coevolutionary ability successfully survive over END period and expand through forming clusters in EXP period for enough higher learning ability of defectors. However, the rule of only defectors have the right of coevolution leads defectors possessing absolute advantage to plunder cooperators in a great extent. Thus, cooperators can rarely survive for they fail to enter EXP period ultimately.

## Discussion

To discuss, we study the environment induced coevolution of learning ability in the prisoner’s dilemma game. In our model, a player can enhance (weaken) its learning ability if its payoff is less than (no less than) the average payoff of its neighbors. In order to investigate the role of heterogeneous learning ability on the evolution of cooperation, we also consider three related rules, where the evolutionary rules are affected by both cooperators and defectors (rule I), only cooperators (rule II) and only defectors (rule III). Quite interestingly, we find the flourishing cooperation in rule II, whereas the bad cooperation behavior in rule I and rule III. Characteristic snapshots reveal that the learning ability of boundary cooperators is always lower than boundary defectors, thus defectors are more willing to change its strategy, further lead cooperators have a higher probability to survive in the enduring period and introduce enhanced network reciprocity into the system. For the cases of rule I and rule III, the situation is the opposite. From previous understanding, the effect of heterogeneity on cooperation depends on specific definition of heterogeneity and updating rules. In this paper, our results show more diverse phenomena. Although the heterogeneity of player’s learning ability in these three rules is introduced via our coevolution rule, the performance of cooperation is totally different. From characteristic snapshots, we argue that heterogeneity alone cannot explain the flourishing cooperation phenomenon, the key lies in the two periods suggested in refs^49,50^. In the END period, cooperation must endure the exploit of defection and begin to form the compact cooperative clusters to support each other. If cooperation can survive in that period, then they can expand their territory in the EXP period to stable state with the support of network reciprocity. These two periods give us a useful analytical framework to explain the promotion or reduction of cooperation. In our results, the higher survival probability is the direct benchmark of the enhanced network reciprocity. In addition, we also implement the same procedure on scale-free network and find that the value of increment *d* has rare impact on the evolution of cooperation, for simple, here, we don’t present the results. We further consider that the payoff comparison only happens among players having the same or different strategies during the process of learning ability updating [refer to Appendix for details].

In our previous investigation^51^, we performed the same procedure in interdependent networks, and found that the promotion of cooperation was derived from the species diversity and the enabled equality. In this paper, the lacked species diversity induced by single network leads cooperation to extinction in rule I, which is consistent with our previous findings. We thus provide a deeper understanding of the evolution of cooperation especially from the viewpoint of heterogeneity.

## Methods

In the present work, we propose a coevolution model on a *L*^2^ square lattice with periodic boundary conditions, where each player located at each node of the network is designated either as a cooperator (C) or defector (D) with equal probability initially. We resort to the weak *PDG* as a pairwise interaction model. Here, defector yields the temptation *T* = *b* while its adjacent player with payoff *S* = 0 chooses to cooperate, cooperators and defectors can yield the reward *R* = 1 and the punishment *P* = 0 when encountering neighbor with the same strategy, whereby 1 < *b*≤ 2 ensures a proper payoff ranking T > R > P ≥ S^9^. And such an arrangement can meet all relevant requirements of general *PDG*.

Irrespective of the initial strategy, the game iterates in accordance with the Monte Carlo (MC) simulation procedure and comprises the following elementary steps. First, a randomly selected player *x* acquires its payoff *P*_*x*_ by summing payoffs from playing game with its four nearest neighbors. Simultaneously, its neighbors can also acquire their payoffs in the same way. Then player *x* decides whether to adopt the strategy *s*_*y*_ from one randomly selected neighbor *y* with payoff *P*_*y*_ via Fermi function^52^:

1 $$W({s}_{y}\to {s}_{x})={w}_{x}\frac{1}{1+\exp [({P}_{x}-{P}_{y})/K]},$$

where *K* = 0.1 depicts the uncertainty of strategy adoption^53^. In general, players with higher payoff are more likely to spread their strategies. Moreover, *w*_*x*_ characterizes the strength of learning activity of player *x* and evolves after strategy adoption procedure at each MC step.

To ensure the fairness of the game, each player is endowed with the maximum learning ability *w*_*x*_ = 1 in the beginning. The heterogeneity of learning ability is caused by updating the value of *w*_*x*_ with an adjustable factor *d* (0 ≤ *d* ≤ 1):

2 $${w}_{x}=\{\begin{array}{cc}{w}_{x}+d, & {\rm{if}}\,{P}_{x} < {\bar{P}}_{x},\\ {w}_{x}-d, & \,{\rm{if}}\,{P}_{x}\ge {\bar{P}}_{x}.\end{array}$$

Here, ${\bar{P}}_{x}$ describes the average payoff of the environment in which player *x* is located, we measure it by function:

3 $${\bar{P}}_{x}=\frac{{\sum }_{i=1}^{{k}_{x}}\,{P}_{i}}{{k}_{x}},$$

where *P*_*i*_ denotes the payoff of the *i*-th neighbor and *k*_*x*_ represents the number of neighbors (degree) of the player *x*. Here, we fix the minimum learning ability *w*_*x*_ = 0.1 for avoiding frozen states. Intuitively, our model turns to a classical homogeneous system (traditional version) if *d* = 0.

One full Monte Carlo (MC) step involves all players having a chance to update their strategy and learning ability on average. The density of cooperation *f*_*C*_ reaches its stationary state after a sufficiently long relaxation time within 5 × 10^4^ to 10^5^ full MC steps and is determined by averaging in the last 5 × 10^3^ steps. Depicted results were obtained on populations varied from *L* = 100 to 400 and were averaged over 20 independent realizations to further improve accuracy.

## Supplementary information

Supplementary Info
