# Supplementary material for: The influence of heterogeneous learning ability on the evolution of cooperation
Source: Sci Rep. 2019 Sep 26;9:13920. doi: 10.1038/s41598-019-50451-2 (PMC6763452; doi:10.1038/s41598-019-50451-2)
Supplement: Supplementary file 1 — Supplementary Info [file 41598_2019_50451_MOESM1_ESM.pdf]

The influence of heterogeneous learning ability on the evolution of cooperation

Xiaogang Li[1], Yini Geng[1], Chen Shen[1], and Lei Shi[1,*]

1 School of Statistics and Mathematics, Yunnan University of Finance and Economics, Kunming 650221, China.

* Corresponding author: shi_lei65@hotmail.com

The influence of heterogeneous learning ability on the evolution of cooperation

Xiaogang Li[1], Yini Geng[1], Chen Shen[1], and Lei Shi[1,*]

1 School of Statistics and Mathematics, Yunnan University of Finance and Economics, Kunming 650221, China.

* Corresponding author: shi_lei65@hotmail.com

Appendix

We further explore the effect of heterogeneous learning ability. In detail, the payoff comparison during the process of updating learning ability only happens among neighbors having the same or different strategy in Fig. A1 and further show characteristic snapshots of strategies and learning ability under specific initial distribution in Fig. A2 and A3.

In Fig. A1, the left and right panels depict the cases that payoff comparison in the process of updating learning ability only happens among players having the same and different strategy. Apparently, cooperation is significantly inhibited in the left panel for f_C decreases monotonously and rapidly with the increase of d, especially when $d = 0.03$, f_C drops to 0 even if b is pretty small. However, the situation gets complicated when the payoff comparison is limited to players having different strategy. For small d ($d = 0.1$ and 0.2), cooperation can be facilitated with high temptation to defect b. The rest values of d perform the same effect on cooperation with d in the left panel.

For further exploring the internal process of evolution for above two mechanisms, we present evolutionary snapshots of strategy (the top row) and learning ability w (the bottom row) at the same MC steps under specified initial distribution in Fig. A2 and A3. Similar to the case in Figure 5, defectors with lower learning ability and higher payoff disintegrate cooperation camp rapidly in END period shown in Fig. A2, which leads to

the extinction of cooperators eventually (not showed in this figure). In Fig. A3, players can only compare payoff with neighbors possessing different strategy. On the contrary, cooperators with coevolutionary ability successfully survive over END period and expand through forming clusters in EXP period like the process in Figure 4.

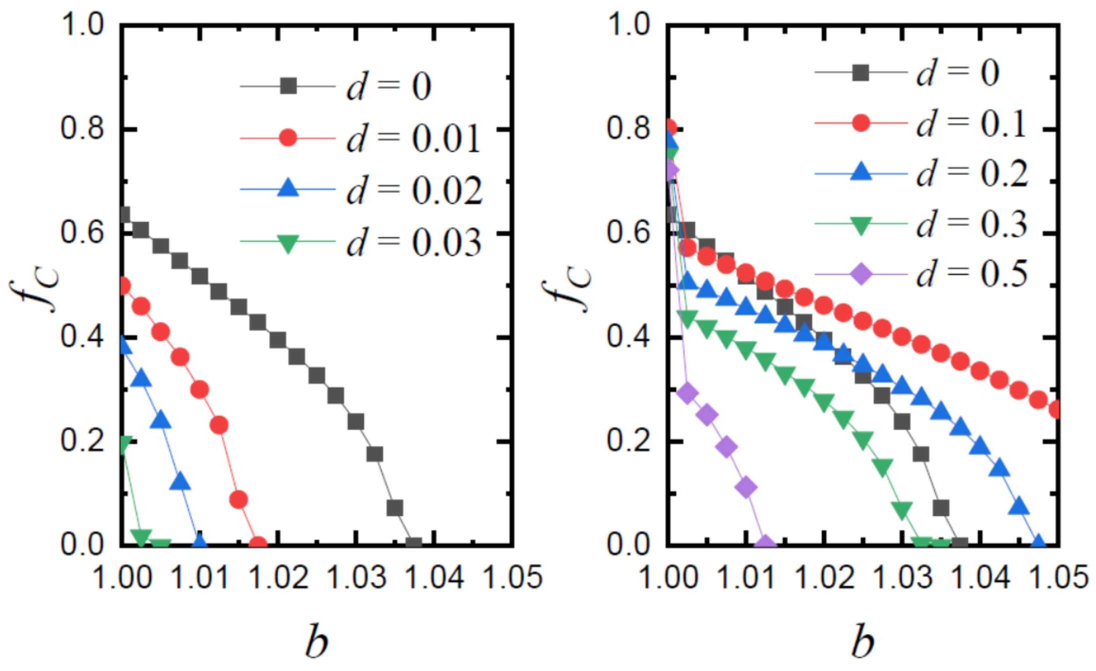

Fig. A1. Density of cooperation f_C in dependence on b for different d. The left (right) panel corresponds when the payoff comparison only happens among players having the same (different) strategy.

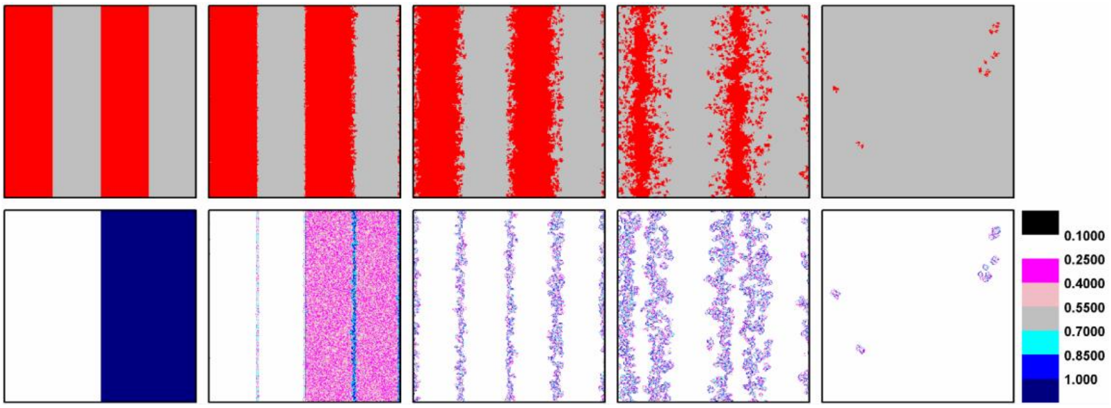

Fig. A2. Characteristic snapshot of strategy (the top row) and learning ability (the bottom row), when the payoff comparison only happens among players having the same strategy under specific initial distribution. The results are obtained for $d = 0.02$, $b =$

1.02 and $L = 300$.

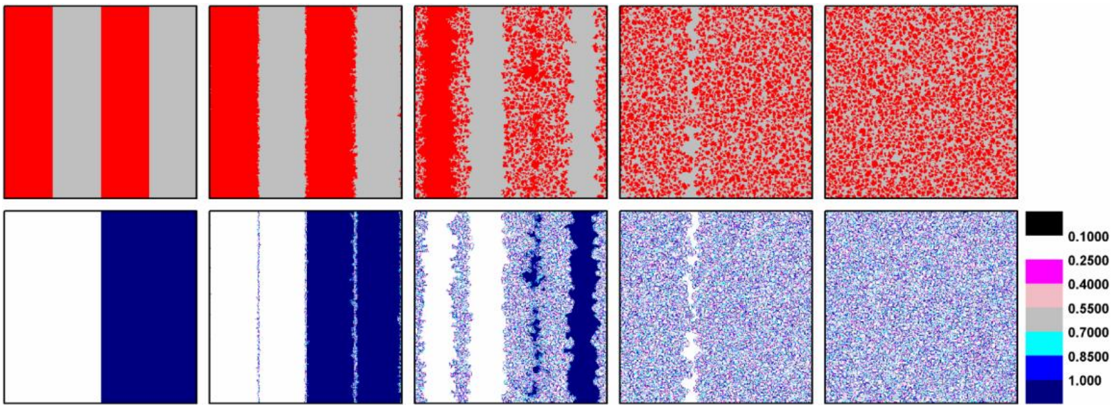

Fig. A3. Characteristic snapshot of strategy (the top row) and learning ability (the bottom row), when the payoff comparison only happens among players having the different strategy under specific initial distribution. The results are obtained for $d = 0.1$, $b = 1.02$ and $L = 300$.
